# Mr. Thelwall, on Difficulty of Speech

**Published:** 1806-02

**Authors:** John Thelwall

**Affiliations:** Brownlow-Hill, Liverpool


### To Dr. BATTY.

Sir,

As prior to the time of Dr. Darwin, Dr. Wallis is, I believe, the only modern grammarian who has treated the subject of human speech anatomically, (unless, indeed, old Ben Jonson may, in some respects, be regarded as an exception) the following prescription may, at least, be regarded as a curiosity worthy of some notice; and the example of that learned physician may, perhaps, have a beneficial operation in recommending the study and treatment of impediments of speech, as a branch of medical science.

The document was communicated to me by Dr. Briggs, (then of Kendal) during my residence in that town; and, perhaps, I cannot more efficiently further the object of assisting the progress of useful information, which the kindness of the communicator had evidently in view, than by laying it, with some little commentary, before the world.

" *Dr. Wallis's Directions for Mr. Thomas Wood, to Help*
" *his Speaking.*"

*Towcester, May* 24, 1687.

" The chief cause of his difficulty in speaking I take to be, that *his tongue is somewhat too long*, and there is too much of it to speak plainly, unless it be carefully managed, for which these directions are proper.

" For pronouncing *m, b, p,* let him take care not to press his lips too hard, but let them only touch softly; and keep down his tongue, not by pulling it back into his throat, but keeping it flat in his mouth.

" The like for *v, f, ph, w, wh;* save that for these, a little space is to be left between his lips for the breath to pass.

" For *n, d, t, th, s, z, ja, ge, dge, ce, che, sh, l, r,* which are to be pronounced by the tongue; let him put up only the tip of his tongue (not the middle of it) to the roof of his mouth, near the root of the upper teeth, and keep down the middle of it.

" For *k, ca, ga, ha, y, x,* or what else is to be formed in the throat, let him take care not to draw back the tongue too much into the throat, but only touch gently with the hinder part of it; and let him speak leisurably; not not too fast, and with his mouth well opened. And with care and heed to practice these rules, he will in a short time speak much better. JOHN WALLIS."

What the efficacy of this prescription was I know not; nor can the propriety of the directions be pronounced upon with any absolute decision, unless it were possible to be minutely informed of the particular phenomena of the case, as to utterance, conformation, and physiognomy. Upon the whole however, as general rules, so far as they go, they may certainly be regarded as judicious. The general principle, "let him take care not to press his lips too hard" for the labial sounds, and "let him take care not to draw back the tongue too much into the throat, but only *touch gently* with the hinder part of it," for the guttural, is excellent; and shews that Dr. W. was not unacquainted with the mistaken habit of voluntary action, out of which impediment very frequently arises. Equally important is the hint about "speaking leisurably." *Take time, and keep time,* properly comprehended, comprises almost the whole mystery of elocutionary attainments: but, alas! how much of science does the full comprehension of the latter clause of this short sentence presuppose, and how little is the subject understood among us! Against the direction which follows, however, "*and with his mouth well opened,*" as a general regulation, I enter my public and solemn protest. Never yet was wide-mouthed speaking either facile or harmonious; and for one impediment or impropriety of utterance that has been removed (if ever one was) by the vulgar exclamation, "*open your mouth!*" I am convinced that fifty have been produced. The lips indeed must be sufficiently parted, and the aperture so modified as to give proper egress to the respective sounds; and, at the same time, without any pouting protrusion, must be so disentangled from the teeth as not to interfere with their vibrations—so essential both to the tone of the voice and the distinctness of enunciation: but let the speaker, who knows the value of that axiom which enjoins us to produce the greatest quantum of effect by the smallest practicable exhaustion of physical power, beware of the widely opened mouth. I have just dismissed two pupils (ladies of this town) with the object of my instructions, I trust, in a considerable degree, accomplished; and whose impediment seems to have been principally attributable to this very maxim.

It should be observed, also, that though there are certain tain conformations of mouth, with respect to the position of the lower jaw, which would render the direction to elevate the tip of the tongue *towards* the roof of the mouth, for the formation of *s, z, ch,* and *sh,* yet, as a general rule, it will by no means hold; and how any person could pronounce *th* in the manner prescribed, I am utterly at a loss to conceive. In all directions for the position of the tongue in the formation of the lingual elements, great attention should undoubtedly be paid to the anatomical conformation of the particular mouth in question. Dr. Darwin, in his elaborate and valuable note upon this subject, affixed to his Temple of Nature, through the want of comparing a sufficient number of facts, has dictated as *universal laws* for the formation of certain of the elements, positions of the tongue, in which, in my own instance, it would be utterly impossible to produce them; shall I be pardoned for going still further, and saying, that I suspect that some portion of Dr. D's own impediment, such as I remember it to have been, might be accounted for from some errors in his own theory of the anatomy of enunciation. Another precaution of extreme importance in the management of impediments, is clearly to ascertain the seat of the defect; whether it be labial, lingual, or guttural; or whether in the primary organs of vocal impulse. To this Dr. Wallace does not appear to me to have sufficiently attended. He considers the chief cause of the difficulty to have been the tongue being too long, and there being too much of it; and yet the part of the impediment to which his first attention is directed must have been purely labial; and, indeed, though the tongue in customary language and popular prejudice, be blamed for all, the lips are much more frequently the guilty organ. My experience of cases of impediment has not much increased my faith in the vast differences and caprice of Nature in the conformation of tongues. That some tongues want more regulation and government than others; that some are larger, and others smaller, cannot be denied; but, that the mere fashion and quantity of the tongue, independently of aukward tricks and habits, is a frequent cause of impediment, I do not believe.

Another discrimination which it is necessary to make, comes immediately within the sphere of the medical practitioner. Impediments are frequently complicated with what are called nervous affections; from which sometimes they spring, and which sometimes they considerably aggravate. To ascertain in such cases the exact boundaries between between mental and physical causation—to determine how far the habit has resulted from the disease, and how far the disease has been produced by the habit, is perhaps more desirable than practicable; yet science may do something towards it; and I have certainly seen many cases that have made me wish to see much more frequently, that union which Dr. W. exhibited of elocution and medical science.

*Brownlow-Hill, Liverpool,*

*Dec.* 20, 1805.

JOHN THELWALL.

